# Phages from Ganges River curtail in vitro biofilms and planktonic growth of drug resistant *Klebsiella pneumoniae* in a zebrafish infection model

**DOI:** 10.1186/s13568-021-01181-0

**Published:** 2021-02-15

**Authors:** Niranjana Sri Sundaramoorthy, Subramaniam Thothathri, Muthumeenakshi Bhaskaran, ArunKumar GaneshPrasad, Saisubramanian Nagarajan

**Affiliations:** Center for Research in Infectious Diseases (CRID), School of Chemical and Biotechnology, SASTRA Deemed University, Tamil Nadu, Thanjavur, 613401 India

**Keywords:** Bacteriophage, *Klebsiella* spp., Phage therapy, Ganges river

## Abstract

Bacteriophages are a promising alternative for curtailing infections caused by multi drug resistant (MDR) bacteria. The objective of the present study is to evaluate phage populations from water bodies to inhibit planktonic and biofilm mode of growth of drug resistant *Klebsiella pneumoniae *in vitro and curtail planktonic growth in vivo in a zebrafish model. Phage specific to *K. pneumoniae* (MTCC 432) was isolated from Ganges River (designated as KpG). One-step growth curve, in vitro time kill curve study and in vivo infection model were performed to evaluate the ability of phage to curtail planktonic growth. Crystal violet assay and colony biofilm assay were performed to determine the action of phages on biofilms. KpG phages had a greater burst size, better bactericidal potential and enhanced inhibitory effect against biofilms formed at liquid air and solid air interfaces. In vitro time kill assay showed a 3 log decline and a 6 log decline in *K. pneumoniae* colony counts, when phages were administered individually and in combination with streptomycin, respectively. In vivo injection of KpG phages revealed that it did not pose any toxicity to zebrafish as evidenced by liver/brain enzyme profiles and by histopathological analysis. The muscle tissue of zebrafish, infected with *K. pneumoniae* and treated with KpG phages alone and in combination with streptomycin showed a significant 77.7% and 97.2% decline in CFU/ml, respectively, relative to untreated control. Our study reveals that KpG phages has the potential to curtail plantonic and biofilm mode of growth in higher animal models.

## Introduction

*Klebsiella pneumoniae* is a Gram-negative nosocomial pathogen that can cause wide range of infections like pneumonia, upper respiratory tract infections, wound infections, diarrhea, urinary tract infection, bacteremia and septicemia (Jarvis et al. 1985). It is one of the leading causes of mortality and morbidity in bacterial sepsis (Vading et al. 2018). Roughly, 54% of *K. pneumoniae* strains that caused neonatal sepsis were observed to be multi drug resistant. As per WHO’s antimicrobial resistant pathogens’ list, carbapenem resistant *Enterobacteriaceae* falls under critical priority category (2017). Thus, there is an urgent need to devise new antimicrobials/resistance modulatory agents against Carbapenem resistant *K. pneumoniae* (CRKP). Due to evolutionary selection pressures, bacteria would invariably gain resistance to either existing or any novel antimicrobial agents/ even for resistance modulatory agents. In such a scenario, lytic phages would serve as a better choice, since; bacteriophages would serve as self-amplifying antimicrobial agents and development of resistance by bacteria against phages is a relatively manageable event with phage cocktail targeting different receptors than the development of antibiotic resistance (German and Misra 2001; Chan et al. 2016; Monferrer and Domingo-Calap 2019; Burmeister et al. 2020).

Phage therapy has been in vogue from 1930 until date, as evidenced by works from Eliava Institute of Bacteriophages Microbiology Virology (EIBMV) at Tbilisi, Georgia (Summers 2012). Commercial production of bacteriophages specific against *Staphylococcus* spp., *Streptococcus* spp., *Pseudomonas* spp., *Proteus* spp., *Shigella* spp. were done till mid 1950′s at EIBMV, HIEET, Poland (Sulakvelidze et al. 2001). Discovery of antibiotics, which exerted a broad spectrum of activity against bacteria and concern regarding use of bacterial viruses for therapy, led to decline in widespread use of phages for therapeutic purposes. Phages for human therapy to treat various ailments like skin infections, wound prophylaxis, burn wounds, respiratory infections and sepsis has been in practice for nearly 90 years (Abedon et al. 2011).

Phages specific for *K. pneumoniae* has been reported previously. Caecal filtrate of a healthy woman undergoing colonoscopy was shown to harbor lytic phage specific to *K. pneumoniae* sub spp.* pneumoniae.* The phage belonged to *Siphoviridiae* that harbored rosette like tail tip with depolymerase activity against capsular type K2 antigen and based on genome sequence analysis, it was classified under novel Kp36like virus (Hoyles et al. 2015). A lytic phage vB_KpnM- Teh.1 (*Myoviridae*), isolated from urban wastewater, was administered intraperitoneally to a BALB/c mice, immediately after an intranasal inoculation of *K. pneumoniae*, which resulted in a significant 7 log reduction of bacterial bioburden (Sasani and Fereshteh Eftekhar 2020).

The objective of the present work is to study the efficiency of *Klebsiella spp* specific phages isolated from Ganges water, in curtailing drug resistant *K. pneumoniae,* both in planktonic and biofilm mode of growth, and also to test efficiency of lytic phages alone and in combination with antibiotic to mitigate in vivo infection using zebrafish as a model before proceeding to higher animal models. Towards this goal, in the present study, *K. pneumoniae* specific phage was isolated from Ganges water and its efficiency in curtailing planktonic and biofilm mode of growth in a drug resistant clinical isolate (MTCC 432) was evaluated both in vitro and in vivo in a zebrafish infection model.

## Materials and methods

### Bacterial Strain and other materials

*K. pneumoniae* sub spp. *pneumoniae* (MTCC 432)*,* an isolate from human urinary tract, was obtained from MTCC, IMTECH Chandigarh. The strain was grown in nutrient broth at 37 °C and preserved as glycerol stock at − 80 °C. Antimicrobial profiling of the strain was performed by microbroth dilution method (Andrews and Andrews 2001). All media used in the study was procured from HiMedia Labs, India. Antibiotics, salts and other chemicals were procured from Sigma-Aldrich, USA or Sisco Reaearch Labs, India.

### Phage isolation and purification

Bacteriophages specific against *K. pneumoniae* was isolated by inoculating an overnight culture of *K. pneumoniae* in the collected Ganges water, supplemented with 10X nutrient broth and incubated for 24 h at 37 °C. The enriched water was centrifuged and the supernatant, containing phages, was collected. The supernatant was filtered through 0.45 µm filter (Whatman, GE Healthcare Life Sciences) and stored at 4 °C until titer determination. The bacteriophage titer was determined as described previously (Adams 1959). Briefly, the phage containing lysate was serially diluted in SM buffer (100 mM Sodium Chloride, 8 mM Magnesium sulphate, 50 mM Tris–cl (pH 7.5)) and 100—300 µl of each dilution was mixed with 100 µl of early log phase of the host bacteria (MTCC 432). After incubation at 37 °C for 20 min, the phage-host suspension was mixed with 5 ml of soft agar (0.7% Luria Bertani Agar) and overlaid onto Nutrient agar plates, after incubation at 37 °C for 24 h, PFU/ml was determined. Plates showing 1–10 plaques was used to obtain homogenous plaque morphology by triple purification as mentioned earlier (Bonilla et al. 2016). Briefly, a single plaque was picked, resuspended in SM buffer, vortexed and centrifuged. The supernatant was serially diluted, incubated with host and plated as mentioned above for phage isolation. The process was repeated thrice ensuring that plaque morphology remained same during iterative process. The resulting phage lysate from triple purification was ultracentrifuged at 4 °C for 1 h at 30,000 rpm to obtain concentrated, high-titer phage lysate. The phage lysate was preserved as phage banks at 4 °C for further experiments.

### Determination of host range

In order to discern the host specificity of phage, spot test and liquid assay were performed. The phage was tested against different clinical isolates of *K. pneumoniae* (BC936, E474, U2016, BC1415, BC1994) and *E. coli* (U3790, U3176, U1007, U1024, U2354) obtained from Sundaram Medical Foundation, Chennai, India. In addition, the phage was also tested against reference strains of *E. coli* (MG1655), *Acenitobacter baumannii, Enterobacter cloacae, Pseudomonas aeruginosa* and *Enterococcus faecalis.* Spot assay was done as reported earlier (Zhao et al. 2019). 300 µl of fresh bacterial culture was added to soft agar, overlaid on nutrient agar plate and allowed to solidify. 5 µl of the purified phage lysate (10^12^ PFU/ml) were spotted on to the plate and incubated at 37 °C overnight. For liquid culture based evaluation, 180 µl of culture (0.05 OD) was mixed with phage (10^8^ PFU/ml) and incubated at 37 °C (Xie et al. 2018). The bacterial growth was monitored by measuring absorbance at 595 nm at 0, 30, 60, 90, 120 and 240 min using Tecan Infinite® F50 Microplate reader (TECAN, Männedorf, Switzerland) and the growth curve was plotted. The experiment was carried out in triplicates.

### One Step growth curve

A one-step growth curve of the phages was performed as reported earlier (Pajunen et al. 2000). The host bacteria was grown until early log phase (0.4 OD_595_), centrifuged and resuspended in SM buffer. This was mixed with phages at a multiplicity of infection (MOI) of 1 and was allowed to adsorb for 5–10 min at 37 °C. The mixture was then centrifuged, pellet was resuspended in nutrient media and incubated at 37 °C. Samples were withdrawn for every 5 min and were plated to determine phage titer. The experiment was performed in triplicates, and values depict the mean of three observations ± standard deviation. Burst size was calculated as the ratio of average PFU/ml in latent period to the average PFU/ml of last three time points. The experiment was done in triplicates and the error bar represents standard error of the mean.

### pH Sensitivity of the isolated phage

The pH stability of the phage and host specificity was evaluated as mentioned previously (Anand et al. 2020). The phages (10^7^ PFU/ml) were incubated in buffers with varying pH (3.0, 5.0, 7.0, 9.0 and 11.0) for 1 h. Post incubation, the phage titer was determined by agar overlay method and the average percentage of surviving phages was calculated from three independent experiments.

### Transmission electron microscopy (TEM)

5 µl of high titer (10^10^ PFU/ml) phage suspension was deposited on a carbon-coated copper grid and were allowed to adsorb for 1 min. Phage particles were stained with 2% aqueous solution of uranyl acetate. Grids were examined with a FEI transmission electron microscope (Model JEM 2100F Jeol, Japan).

### Time kill assay

Efficiency of the phages to curtail bacterial growth *in-vitro* over a time course of 24 h in broth culture was discerned using the time kill assay (Grillon et al. 2016). *K. pneumoniae* at a cell density of 10^6^ CFU/ml was inoculated into LB broth and *Klebsiella *spp. specific monophage (10^8^ PFU/ml) was introduced into the culture. At different time points 0, 2, 4, 6 and 24 h, samples were withdrawn and cells were plated on LB agar and plate counts were determined 24–48 h post incubation. Phage untreated culture was maintained as growth control. The experiment was performed in triplicates. Decline in bacterial counts relative untreated control would imply lytic potential of phages.

### Evaluation of phage-antibiotic combination

The activity of phages in combination with various antibiotics—Streptomycin, meropenem, colistin, erythromycin, ciprofloxacin and tobramycin, were tested by microbroth dilution method. The antibiotics were serially diluted from 64 µg/ml to 1 µg/ml and 10^8^ PFU/ml of KpG phages were added onto the wells. 0.05 OD (~ 10^6^ CFU/ml) of MTCC KP was inoculated and incubated overnight at 37 °C. After incubation, optical density at 595 nm was measured using Tecan Infinite® F50 Microplate reader. The ideal combination of phage and antibiotic, which effectively inhibited bacterial growth was selected and time kill study was performed as mentioned earlier. All experiments were performed in triplicates.

### Crystal violet assay

Biofilms at liquid air interface is akin to biofilms on implantable medical devices (Christensen et al. 1985). Biofilms are formed with *K. pneumoniae* on 96-well micro titer plates with and without phages in Brain Heart Infusion (BHI) broth. Briefly, overnight culture was diluted in the ratio of 1:100 and 100 µl of the diluted culture was added to the wells. 1 h post incubation at 37 °C, KpG phages at different dilutions were added. Appropriate untreated control and broth controls were maintained. 24 h post treatment, biofilms were washed with PBS to remove unbound cells, 125 µl of 0.1% crystal violet was added and unbound crystal violet was washed off. The bound crystal violet, which is an indirect measure of EPS formed was extracted with 30% acetic acid and was quantified by measuring the absorbance at 595 nm.

### Colony biofilm assay

The protocol for forming colony biofilms was essentially as described previously (Merritt et al. 2005). Briefly, 13 mm 0.2 µm membrane filters sterilized by UV radiation placed on sterile BHI agar and inoculated on the center with bacteria or bacteria along with phages. The ability of phages to decrease biofilm formation until 48 h was examined by visual observation.

### Fish toxicity studies

Zebrafish (*Danio rerio*) measuring ~ 4 to 5 cm in length, weighing approx. 300 mg, was purchased from a local aquarium. The protocols adopted were approved by the Institutional Animal Ethics Committee (CPCSEA-510/SASTRA/IAEC/RPP) of SASTRA Deemed to be University, India. Animal acclimatization was performed following previously established protocols (Phillips and Westerfield 2014). The study comprised of two groups: Phage untreated control fish and fish injected with phages. For toxicity evaluation, 6 fish were injected intramuscularly with 10^10^ PFU/ml of phages and mortality of the fish was monitored up to 48 h. At the end of exposure (48 h), fish was sacrificed (anesthetized by 150 mM MS-222 and euthanized by decapitation), liver/brain from two fish of the same group were pooled and homogenized in ice-cold buffer (Tris–HCl, 0.1 M, pH 7.4). The homogenate was centrifuged (10,000*g*, 10 min, 4 °C) to obtain supernatant, which was used for all analyses in duplicates. Brain acetyl-choline esterase and liver carboxyl esterase enzyme activities were determined as reported earlier (Christena et al. 2016). Acteylcholine iodide and α/β naphthyl acetate were used as substrates for determining brain and liver enzyme profiles, respectively. In order to evaluate if the phages induce inflammation or other immunological response, histopathological analysis was performed. Briefly, the muscle/liver tissue of the phage-injected fish were sectioned, subjected to Hematoxylin & Eosin staining, histopathology analysis was performed using a bright field microscope (Nikon Eclipse Ni-U) and was compared with sham control.

### Zebrafish infection

Intramuscular infection of zebrafish with *K. pneumoniae* strain was performed as described earlier (Neely et al. 2002). Briefly, 10 μl (~ 1 × 10^5^ CFU/ml) of *K. pneumoniae* was injected intramuscularly to zebrafish (5/group) using a 3/10-cc U-100 insulin syringe with a 0.5-in.-long, 29-gauge needle. 2 h post infection, phages (10^8^ PFU/ml) were administered intramuscularly. Control fish group injected with *K. pneumoniae* was treated with equal volume of sterile buffer and both the groups were monitored. 48 h post treatment, fish from different groups were euthanized, injected muscle tissue was dissected, homogenized in sterile PBS. The homogenate was serially diluted and plated onto Luria Bertani agar plates and colony counts was determined after 24–48 h of incubation at 37 °C.

### Phage Identification by PCR

Consensus primers for Podoviridae infecting *Klebsiella* species were designed using the PhiSiGns tool (http://phisigns.sourceforge.net) (Dwivedi et al. 2012). To check the consistency of the primers, 60 different genomes of *Podoviridae* infecting *Klebsiella* spp were downloaded. The primers were checked for their ability to bind to the genomes manually. Two primer sets were designed and the sets 1 and 2 corresponds to T4 like DNA helicase/primase and putative DNA primase of reference *Klebsiella* targeting *Podoviridae* phage genomes respectively. The primer sequences are as described in Table 1. 50 ng of phage DNA isolated from cultured plaques was used as template for PCR. No-template controls were maintained. The conditions for PCR amplification are as follows: 95 °C—1 min, 30 cycles of 95 °C—35 s; 54 °C—15 s; 72 °C—1 min and final extension at 72 °C—7 min. The resultant PCR amplicons was sequenced using Sanger sequencer and its identity specifically to viral family *Podoviridiae* was further confirmed by BLAST analysis.

**Table 1:** Details of primers used in the study

Sequence name	Sequence (5′–3′)	Expected product size (bp)	Reference Genome	Location of the expected product in genome	Gene product
CL1_FP	AAGGAGGGAATTACGGGATG	193	KU183006.1 *Klebsiella* phage vB_KpnP_IME205, complete genome	11,337–11,475 bp	T4 like DNA primase/ helicae
CL1_RP	AC(A/G)ATGGAGCCATTCTGGTC				
CL2_FP	GGAATCATGCCTGAAATGGT	125	NC_023567.2 *Klebsiella* phage F19, complete genome	7593–7676 bp	Putative DNA primase
CL2_RP	ATTACCCACTTCGGTTGCTG				

## Results

**Fig. 1:**
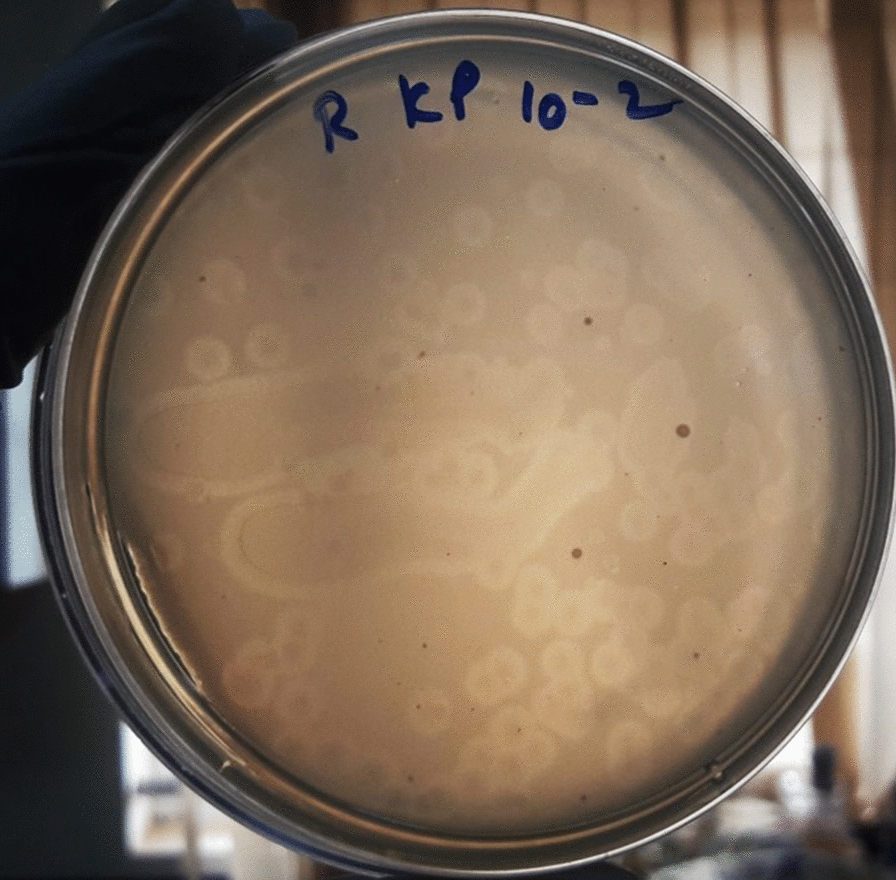
Plaque morphology of the *Klebsiella pneumoniae* specific phages isolated from Ganges river (KpG) The Ganges water from Rishikesh was enriched with the host and plated by agar overlay method. KpG shows depolymerase activity, which is evident from the halo around the plaques

**Fig.2:**
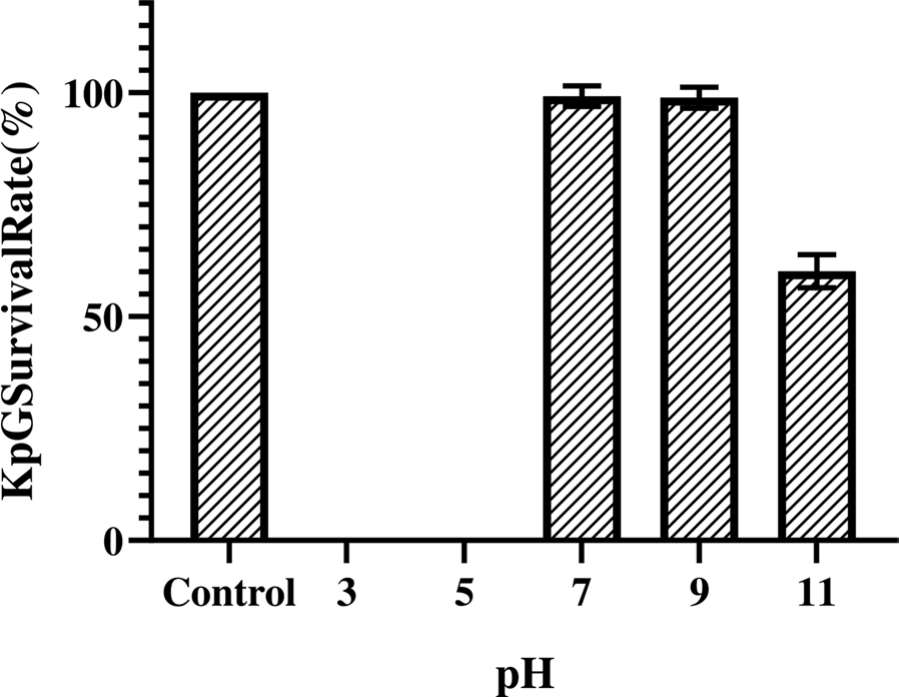
Stability of KpG at different pH. 10^7^ PFU/ml of purified phage lysate was incubated in buffers of varying pH 3.0, 5.0, 7.0, 9.0 and 11.0 for 1 h. Phage titer was estimated using agar overlay method and survival percentage was calculated using phage titers measured in SM buffer (pH 6.8) as control. The experiment was performed in triplicates

Ganges river water was sampled at two different locations Haridwar, and Rishikesh. Attempts to isolate phages from these water sources by agar overlay method showed that only Ganges water sampled from Rishikesh displayed plaques of uniform morphology against MTCC 432 and was designated as KpG (Fig. 1). Although other sample from Haridwar displayed a lysis zone, it did not display distinct plaque morphology that could be further purified. Lack of phages from different water sources, for a ubiquitous strain like *K. pneumonia*, was rather uncommon. Hence, we checked whether the strain used in the present study produced capsule. Staining revealed that MTCC strain indeed produced capsule (Additional file 1: Fig. S1). Morphology of plaques from Ganges water showed a halo around the clear zone, which might indicate depolymerase activity of KpG that could target the capsule of the strain (Fig. 1) (Domingo-Calap et al. 2020). The phage displayed stability at pH: 7.0, 9.0, 11.0 with a survival rate of 99%, 98% and 60% respectively (Fig. 2). Interestingly, we observed that plaques from pH 11.0 group did not display even a faint halo around the lysis area, indicating the inability of these phages to produce depolymerase at pH 11.0 (Additional file 1: Fig. S2). In order to check if KpG is specific to its capsulated *K. pneumoniae* host, spot test and liquid assays were performed against diverse bacterial strains viz., other capsulated strains of *K. pneumoniae* (BC936, E474, U2016) and uncapsulated strains of *K. pneumoniae* (BC1415 and BC1994), *E. coli, A. baumannii, P. aeruginosa, E. faecalis, E. cloacae*. We did not observe any lysis in these different bacteria except MTCC 432 strain (Additional file 1: Fig. S3), implying host tropism (specificity) of phages to a particular capsular variant of *K. pneumoniae* (MTCC 432).

**Fig. 3:**
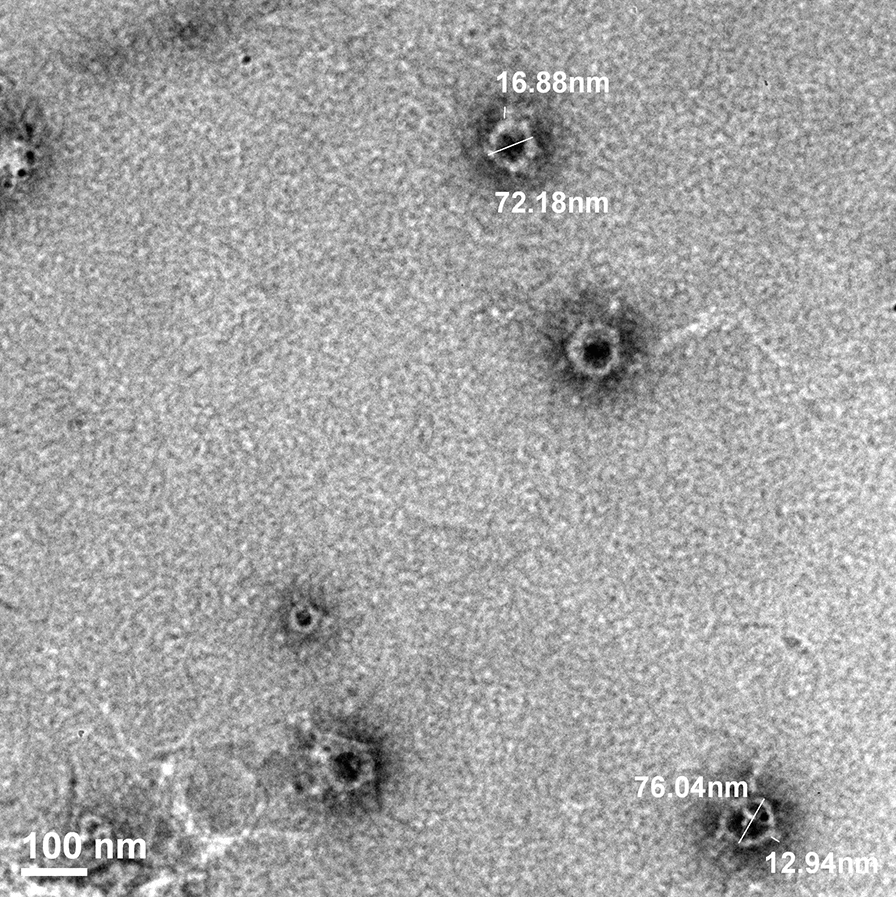
TEM analysis showed that KpG phages belonged to the family *Podoviridae*. Triple purified Phage lysate of high titer was stained with 2% uranyl acetate and visualized under FEI transmission electron microscope (Model JEM 2100F Jeol, Japan). Image presented is representative of multiple images

KpG phage was purified by triple plaque purification method and its PFU/ml were determined. One-step growth curve of KpG phage revealed (Additional file 1: Fig. S4) a larger burst size of 224 PFU/cell and a latent period of 20 min. TEM images of the KpG suggested it belonged to *Podoviridae* (Fig. 3). We designed two PCRs for identification of *Podoviridae* by analyzing the conserved regions of their genomes. We obtained 295 bp and 97 bp amplicons for CL1 and CL2 respectively (Additional file 1: Fig. S5). The PCR amplicons were sequenced by Sanger sequencing and have been submitted in Genbank (Accession numbers: CL1-MW026685 and CL2-MW026686). The sequences were further subjected to BLAST analysis against nucleotide database of NCBI and results from BLAST analysis revealed that CL1 amplicons exhibited a high degree of similarity ((97–98% similarity with e score 1.55e ^−59^) with T7-like phage primase/helicase protein Accession No: MN149903.1; Location: 18,733–18,871 and CL2 amplicons exhibited greater similarity (96.7% similarity with an e score of 5.88e^−29^) with putative DNA primase (Accession No: KP708986.1 Location: 7283 – 7366) with genome sequences of *Klebsiella phages* belonging to *Podoviridae.* Both Morphology by TEM and Sequence similarity with highly conserved region of *Podoviridae* showed that KpG isolate also belongs to family *Podoviridae*.

**Fig. 4:**
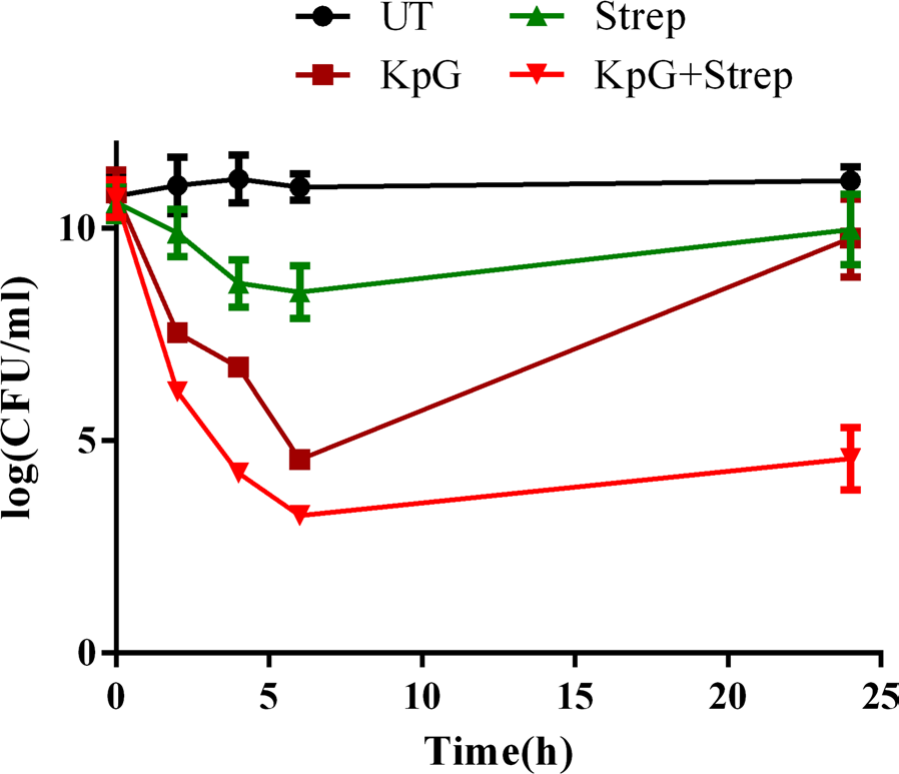
KpG phages in combination with  Streptomycin caused discernible reduction in colony counts in a time kill assay. 10^6^ CFU/ml of *K. pneumoniae* was inoculated along with 10^8^ PFU/ml of KpG phages and Streptomycin (individually and in combination). Samples were withdrawn at 0, 2, 4, 6 and 24 h serially diluted and plated on to LA plates to determine colony counts. Experiments were performed in triplicates and error bar represent standard error of the mean

Time kill studies performed with KpG phages at an MOI of 1, resulted in 0.5 log decline initially but, significant regrowth was obtained at 24 h (data not shown). Hence, time kill curve was performed with a MOI of 100, at which KpG phages caused an initial 3–4 log decline in bacterial cell counts by 4 h (Fig. 4). However by 24 h, regrowth in bacterial cell counts were still observed, which might be due to evolution of phage resistant mutants, which was evident by allowing phages to interact with both phage exposed cells and unexposed cells. At different dilutions tested, phage unexposed cells of MTCC 432 strain was lysed by KpG phage whereas, when the same strain was exposed to KpG phages for 5 h, the cells were resilient to lysis at all dilutions of KpG phage tested (Additional file 1: Fig S6), implying that exposure to the phage for 5 h (~ 10 generations) triggers evolution of phage resistant mutant. Capsular staining of strain by 5 h revealed that strain possessed capsule (data not shown) and inability of phages to lyse could not be attributed to differential expression of capsular polysaccharide. A similar phenomenon was reported earlier (Holst Sørensen et al. 2012).

In order to circumvent the evolution of phage resistant mutant, we tested the phages in combination with different antibiotics (Streptomycin, meropenem, colistin, erythromycin, ciprofloxacin and tobramycin) against *K. pneumoniae*. We found that the combination of streptomycin and phages were effective in inhibiting the growth of *K. pneumoniae,* in addition, phages reduced the MIC of streptomycin by eightfold (from 64 µg/ml to 8 µg/ml) implying synergy of KpG phages with streptomycin. We further evaluated the combination in vitro in a time kill assay. As expected, the combination of streptomycin and KpG caused a 7-log decline relative to the untreated control within 6 h and by 24 h despite a slight regrowth, a decline of 6 log relative to the other groups was maintained (Fig. 4). Streptomycin treatment alone did not show significant reduction in colony counts at 24 h.

**Fig. 5:**
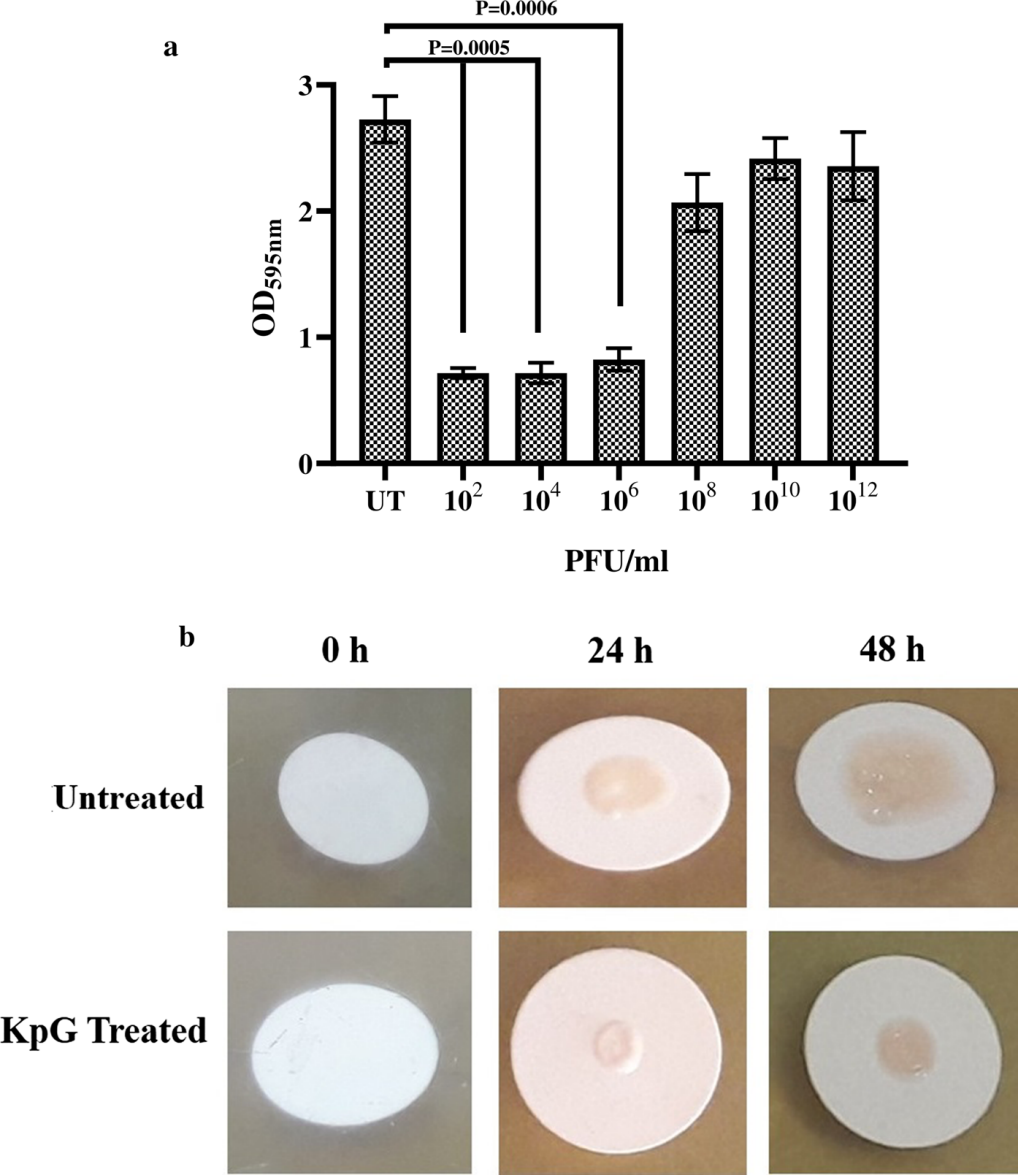
KpG phages efficiently inhibited biofilm formation of *K. pneumoniae* at liquid–air and at solid-air interface. a) Biofilms were formed in micro titer plates with or without KpG phages and washed with PBS. 24 h post treatment, Crystal violet was added and after 15 min, the unbound crystal violet was removed and the stain was extracted by acetic acid and the absorbance was measured at 595 nm, b) Membrane filters were placed on BHI agar and inoculated with bacteria with or without KpG. The ability of phages to decrease biofilm formation can be examined visually till 48 h. Images are representative of three independent experiments

Ability of phages to inhibit biofilms formed at liquid air interface was discerned by crystal violet assay, which showed that KpG phages caused a significant fivefold decrease in biofilm formation at 10^–8^ dilution relative to biofilm formed by untreated bacteria (Fig. 5a). *K. pneumoniae* can also cause wound infections in immune compromised persons, wherein it typically forms biofilms at solid air interface, in order to mimic biofilms at solid air interface, colony biofilms of *K. pneumoniae* were formed and ability of phages to inhibit colony biofilms were evaluated. The results revealed that treatment with KpG phages caused a substantial decline in colony biofilm formation at 24 and 48 h relative to the untreated control (Fig. 5b). Thus KpG phages displayed potent biofilm inhibitory activity against MTCC 432 strain of *K. pneumoniae*.

In order to discern whether biofilm inhibitory effect of phages could be attributed to lytic potential of phages, live dead imaging was performed on phage treated biofilms. *K. pneumoniae* were allowed to form biofilms on glass slides and were treated immediately with KpG phages and 24 h post treatment, the unbound cells were washed and slide was stained with live/dead imaging kit as per manufacturer’s protocol and was imaged using Nikon Fluorescent microscope (Nikon Eclipse Ni-U, Nikon, Japan). Live/Dead imaging showed that treatment with KpG phages caused substantial proportion of dead cells as evidenced by yellow cells in merged image indicative of dead cells and only a small sub population were alive indicating lytic potential of phages (Additional file 1: Fig. S6).

KpG phages were injected into zebrafish and toxicity due to phages were evaluated by assessing liver (alpha and beta naphthol) and brain (acetyl choline esterase) enzyme profiles of zebrafish. Phage administration did not cause any significant variation in liver enzyme profiles of zebrafish. But, discernible increase in brain acetyl choline esterase profiles were observed, which was not statistically significant (P = 0.0529) (Additional file 1: Fig. S7). Hematoxylin and eosin stained muscle and liver tissue from KpG phages treated group relative to phage untreated group also revealed that both muscle and liver tissue appeared normal and was similar to untreated control (Additional file 1: Fig. S8) implying that phage administration did not induce either morphological/biochemical aberrations in zebrafish.

**Fig. 6:**
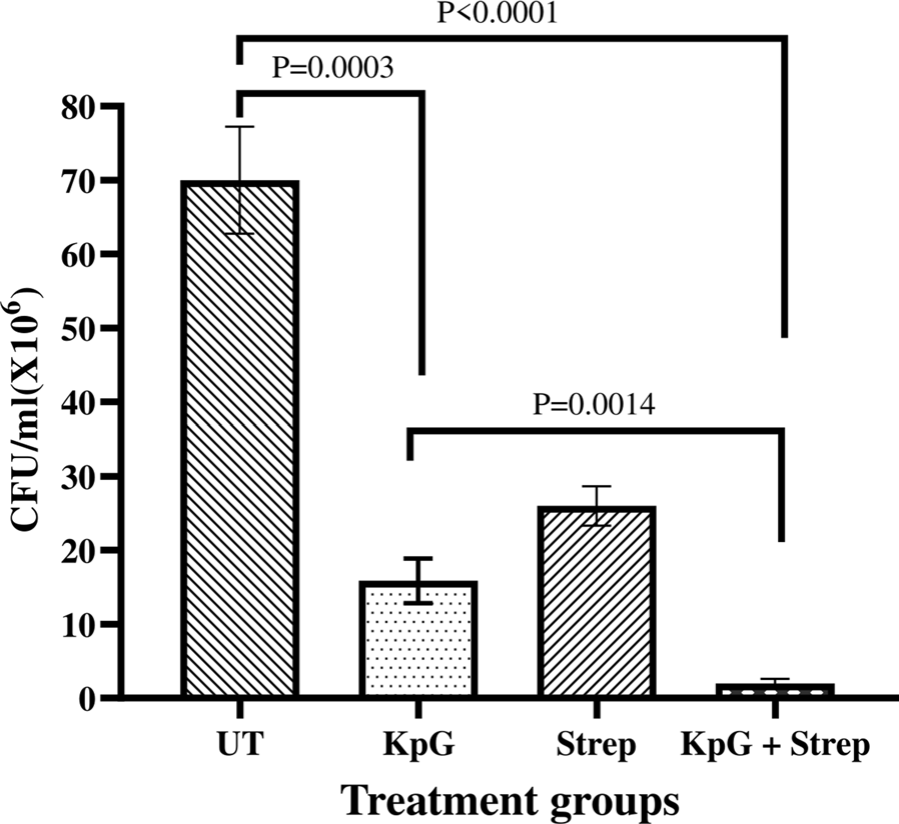
KpG phages reduced bacterial bioburden in infected fish. 10 µl of *K. pneumoniae* (10^6^ CFU/ml) were injected intramuscularly in zebrafish and 2 h post infection, KpG was administered intramuscularly. 24 h post infection, the muscle was dissected, serially diluted and plated on to LA plates. CFU/ml was calculated. Experiment was performed in triplicates and error bar represent the standard error of the mean

Finally, to discern in vivo efficacy of phages, 10 µl of MTCC 432 strain of *K. pneumoniae* corresponding to ~ 1 × 10^5^ CFU/ml was injected into muscle tissue of zebrafish. 2 h post infection, groups of 6 fishes were injected intramuscularly either with Streptomycin, KpG phages (10^8^ PFU/ml) or a combination of KpG phages + Streptomycin and the ability of phages alone/in combination with antibiotics to curtail bacterial growth in infected muscle tissue was evaluated. Our results showed that relative to untreated control, streptomycin treatment caused 62.8% reduction in cell counts (CFU/ml), KpG phages treatment caused 77% reduction in cell counts and a combination of KpG phages + Streptomycin caused 98% decline in cell counts in infected muscle tissue underscoring ability of phage antibiotic combination to significantly reduce bacterial bioburden. All treatments caused statistically significant decline in cell counts relative to untreated control (Fig. 6). This reiterates the fact that lytic phages from Ganges were not only effective in vitro against planktonic and biofilm mode of growth, it was also effective in vivo in curtailing bacterial growth in infected muscle tissue of zebrafish and hence has the potential to mitigate in vivo infection by *K. pneumoniae* in higher animal models.

## Discussion

CRKP pose grave threat to public health especially in immune compromised patients and in neonates where mortality rate is very high (Investigators of the Delhi Neonatal Infection Study (DeNIS) collaboration 2016). CRKP classified as a critical priority pathogen by WHO, which severely limits therapeutic options available (Tumbarello et al. 2012). In this scenario, newer antibiotics are not the way out since bacteria will easily gain resistance to new antimicrobial agents due to evolutionary selection pressures. Although resistance modulators like betalactamase inhibitors, efflux pump inhibitors, quorum sensing inhibitors, cationic peptides are seen as a viable alternative, they were not as effective against MDR pathogens as expected. These bugs could circumvent these agents, for example, by producing betalactamases with metal co-factors that could not be inhibited by betalactamase inhibitors or by expressing redundant efflux transport proteins to extrude the antibiotics etc. Hence, to tackle AMR menace, biological control agents like lytic phages are considered as better alternatives (Aleshkin et al. 2016). The advantage of phage therapy is that it is highly specific and targeted hence it does not disturb commensal microbiota or lead to dysbiosis, given importance of commensal microbes in our health and well-being (Blander et al. 2017; Novince et al. 2017), whereas antibiotics could potentially harm commensal microbes resulting in dysbiosis.

Rivers like Ganges harbor a wide diversity of phages, which affords a rich source of targeted biological control agents against rising menace of drug resistant pathogens. The reason for thriving diversity of phages in Ganges river is that the Himalayan permafrost (source of Ganges river) upon melting, released abiotic phages that were trapped long time ago, contributing to unique diversity of bacteriophages (Khairnar 2016). This rich diversity of phages could be used as a viable source to curtail MDR pathogens in both planktonic and biofilm mode of growth. A previous report has also shown that a cocktail of phages isolated from Ganges river and sewage water against carbapenem and colistin resistant *E. coli*, *K. pneumoniae* and *Enterobacter* spp effectively reduced mixed bacterial load (Manohar et al. 2019).

In our study, the KpG from Ganges water was specific only for one clinical isolate obtained from urinary tract (MTCC 432 strain) and not towards other capsulated or uncapsulated strains of *K. pneumoniae* or *E. coli* or other bacterial species like *P. aeruginosa, E. cloacae* and *A. baumannii*. Similar to our observation, another report showed that four lytic phages, belonging to the family *Podoviridae*, infecting *K. pneumoniae* capsular type K22, were isolated from environmental samples. They possessed narrow infectivity only against a *K. pneumoniae* clinical isolates with K22 capsular type (Domingo-Calap et al. 2020). Moreover, the plaque morphology showed a halo around the lysis area, implying presence of depolymerase that lyses the capsule (Domingo-Calap et al. 2020). Interestingly, MTCC 432 strain produced capsule (Additional file 1: Fig. S1), as we observed a halo around the plaque (Additional file 1: Figs. S2, S3), it is likely that KpG phages possess capsular depolymerases (Oliveira et al. 2019). pH stability studies showed that KpG phages were unstable at lower pH and retained infectivity from pH 7.0 to pH 11.0. Whereas, depolymerase activity as indicated by halo around the plaques was observed only until pH 9.0. Absence of halo at pH 11.0 implies either lack of depolymerase expression or its inactivation. Since not even a faint zone was observed at pH 11.0 we presumed that it is due to former cause rather than the latter, which remains to be confirmed by RT-PCR studies in future. Tropism of phages is a very well-established phenomenon. In an earlier study, a novel PhiKMV like virus infected only *K. pneumoniae* strains with K1 capsule but not the other capsular types. Capsule deleted K1 mutant strains could not be infected by this phage implying that capsule is essential for infection (Lin et al. 2014). On the other hand, broadly specific multi-host bacteriophage ΦK64-1 produced nine different capsular depolymerases, which enabled this phage to infect 10 different *K. pneumoniae* possessing distinct capsular variants and mutants of these depolymerases failed to infect the corresponding strains (Pan et al. 2017). Capsular tropic phages are known in *Klebsiella spp*. Interestingly, a phage FC3-1 trophic to core polysaccharide and O antigen of LPS of *K. pneumoniae* was reported in an earlier study (Tomás and Jofre 1985).

KpG phages had a larger burst size 224 PFU/cell with a short latent period of 25 min. A range of burst size has been reported with *Klebsiella* specific phages for example KPO1K2 displayed a burst size of 140 PFU/ infected cell (Verma et al. 2009). Another study had shown that Phage Z belonging to family *Siphoviridae* gave a burst size of 320 PFU/infected cell. Phage kpssk3 (*Podoviridae*) isolated from sewage, had a very short latent period of 10 min and a larger burst size of 200 pfu/cell, which was able to lyse 25 clinical isolates of CRKP (Shi et al. 2020). Genome analysis of phage kpssk3 revealed that it did not possess any resistance genes, virulence factors and had depolymerase activity towards exopolysaccharide (Shi et al. 2020).

TEM imaging and sequence similarity to conserved T7 like phage primase/helicase and putative DNA primase of *Podoviridae* revealed that the Ganges phages belong to family *Podoviridae*. Most of the reported phages fall under family *Siphoviridae, Podoviridae* or *Myoviridae* (Kęsik-Szeloch et al. 2013)*.* Significant biofilm inhibitory effect (Fig. 5a, 5b) coupled with the fact that MTCC strain produces capsule, imply that depolymerase of KpG phage might be effective against extracellular polymeric substances of biofilms, which remains to be explored in further studies. Significant proportion of dead cells by live/dead imaging (Additional file 1: Fig. S7) imply that timing of addition of phages is important. Concomitant addition of both bacteria and phages to a localized solid surface causes lysis, whereas if bacteria were initially allowed to attach and initiate biofilm formation, subsequent addition of KpG phages after 1–2 h of bacterial interaction with glass surface did not cause significant bacterial lysis. Previous report has also shown that intranasal administration of phages 3 h prior infection or immediately after infection rescued mice from respiratory infection caused by *K. pneumoniae* strain whereas even 6 h delay in phage administration failed to rescue the mice highlighting importance of timing of phage administration (Chhibber et al. 2008). In an elegant study, to tackle mixed species biofilm formed by *K. pneumoniae* and *Pseudomonas aeruginosa*, wherein, *Pseudomonas spp* forms the bottom layer of the biofilm and is shielded by *K. pneumoniae.* The authors have used a combination of phages especially one that produces *K. pneumoniae* depolymerases, which disrupts the top layer and allows *Pseudomonas aeruginosa* specific phage to access inner biofilm layer. *Klebsiella spp* specific phages lacking depolymerase activity were unable to provide access for *Pseudomonas *spp. trophic phages. Lytic activity of phages was further enhanced in the presence of xylitol (Chhibber et al. 2015).

Among in vivo models, mice is commonly used to evaluate efficacy of bacteriophages in mitigating various infections ranging from burn wound, respiratory infections, sepsis and UTI (Capparelli et al. 2007; Malik and Chhibber 2009; Verma et al. 2009; Cao et al. 2015; Basu et al. 2015). To the best of our knowledge, zebrafish has not been evaluated as an animal model to evaluate bacteriophage therapy in infected fish. The advantages of zebrafish model is ease of availability and maintenance, optical clarity of embryo/larvae, short duration for study, a high degree of genetic homology with humans (Lieschke and Currie 2007). We have shown that injection of *Klebsiella spp* specific phages into adult zebrafish did not pose any toxicity as evidenced by liver and brain enzyme profiles and by histopathological analysis (Additional file 1: Figs. S5, S6). Earlier studies in mice have similarly shown lack of toxicity due to administration of phages and in addition, bioavailability of phages in various tissues were evident within 6 h post injection and half life of phages in mice was roughly 18 h (Verma et al. 2009). In our study, we observed a significant 77% decline in bacterial cell counts in infected muscle tissue relative to untreated control due to lytic activity of KpG phages. But combination of streptomycin + KpG phage was much more effective in reducing colony counts to 97% relative to untreated control. Previous study showed that in a mice full thickness wound model, when efficacy of phage therapy was compared with combination of gentamycin and silver nitrate to tackle infection by *K. pneumoniae*, it was observed that a single dose of phage as a topical application mitigated colonization of *K. pneumoniae,* whereas even multiple applications of silver nitrate and gentamycin failed to afford such protection (Kumari et al. 2011). In another study, same group showed that induced burn wounds in mice infected with MDR *K. pneumoniae* strain was successfully mitigated, with a significant decrease in bacterial load in blood and peritoneal lavage, when phages were administered either by subcutaneous or intraperitoneal route (Malik and Chhibber 2009). In the present study,  high specificity of isolated KpG phage to a particular capsular strain of MTCC 432,synergy of phage with streptomycin in restricting regrowth in time kill assay, remarkable pH stability of phage from pH 7.0 to 11.0. and significant biofilm inhibitory effect coupled with good in vivo effect in restricting bacterial growth in infected muscle tissue underscore the utility of  KpG phages to curtail bacterial biofilms in vitro and restrict planktonic growth of *Klebsiella *spp. in vivo in zebrafish. Lack of toxicity to phages coupled with ease of performing the experiment indicates zebrafish can indeed serve as an initial in vivo model before evaluating efficiency of phages that could potentially mitigate MDR bacterial infections in higher animal models.

## Supplementary Information

**Additional file 1.** Electronic supplementary information.

## Data Availability

Almost all data generated or analyzed during this study are included in this published article (and its Supplementary Information files). The raw data would be available upon request. KpG (phage lysate) can be obtained by contacting the corresponding author Dr. N. Saisubramanian (sai@scbt.sastra.edu).

## Abbreviations

CFU/ml: Colony forming units/ml
PFU/ml: Plaque forming units/ml
MOI: Multiplicity of infection
TEM: Transmission electron microscopy
PBS: Phosphate buffered saline
BHI: Brain heart infusion media
MIC: Minimum inhibitory concentration
MDR: Multidrug resistant
CRKP: Carbapenem resistant *Klebsiella pneumoniae*
