# Supplementary material for: Phages from Ganges River curtail in vitro biofilms and planktonic growth of drug resistant Klebsiella pneumoniae in a zebrafish infection model
Source: AMB Express. 2021 Feb 15;11:27. doi: 10.1186/s13568-021-01181-0 (PMC7884498; doi:10.1186/s13568-021-01181-0)

Supplementary information:

Phages from Ganges River curtail *in vitro* biofilms and planktonic growth of drug resistant *Klebsiella pneumoniae* in a zebrafish infection model.

Niranjana Sri Sundaramoorthy[a], Subramaniam Thothathri[a], Muthu Meenakshi Bhaskaran[a], Arun Kumar Ganeshprasad[a]* and Saisubramanian Nagarajan[a]*

[a],Center for Research in Infectious Diseases (CRID),School of Chemical and Biotechnology, SASTRA Deemed University, Thanjavur, Tamil Nadu, India

*Jointly communicated
Address for correspondence

Dr.N.Saisubramanian, Center For Research in Infectious Diseases (CRID),
School of Chemical and Biotechnology, SASTRA Deemed University Thanjavur -613401; Email sai@scbt.sastra.edu

Dr.G Arun Kumar, School of Chemical and Biotechnology, SASTRA Deemed University Thanjavur -613401; Email arunkumar@scbt.sastra.edu

Fig. S1. Capsular Staining of MTCC 432 strain of *Klebsiella pneumoniae*. Capsular staining showed that the MTCC 432 (*K. pneumoniae*) did possess capsule.

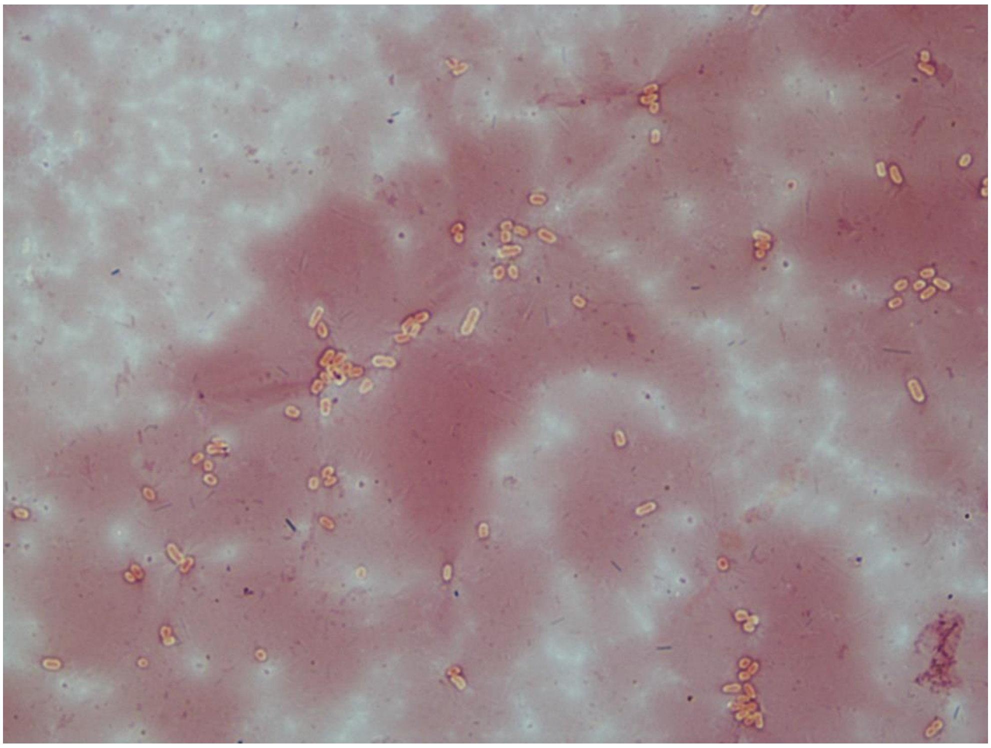

Fig. S2: Effect of pH on KpG phage depolymerase The pH sensitivity test was performed by incubating phages in buffers of different pH – 3.0, 5.0, 7.0, 9.0 and 11.0 for 1 h. After incubation, phage titer was determined by agar overlay method. The experiment was performed in triplicates. Phages were inactivated at pH 3.0 and 5.0. The depolymerase activity was retained at pH 7.0 (a) and pH 9.0. At pH 11.0 (c), the phages remained active but the halo around the plaque was absent, depicting the absence of depolymerase activity at pH 11.0. (Image not to scale)

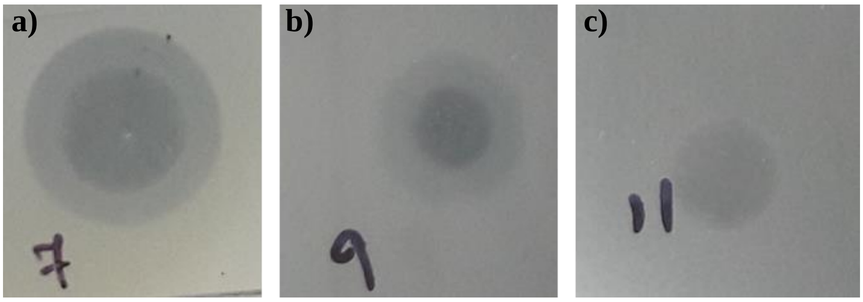

Fig. S3. KpG phages has a narrow host range. KpG was tested against different bacterial strains to determine host specificity. A) Spot test: 300 µl of each bacteria was added to soft agar and poured on to nutrient agar plate. 5 µl of phage lysate was spotted on the plate and incubated overnight at 37°C to check lysis. B) Liquid assay: Diluted culture of 0.05 OD was incubated with 10^8 PFU/ml of KpG for 240 min (3 h). Samples were collected at 0, 30, 60, 90, 120 and 240 min and the absorbance at 595 nm was recorded to check bacterial growth. The experiments were performed in triplicates and the error bar represents the standard error of the mean.

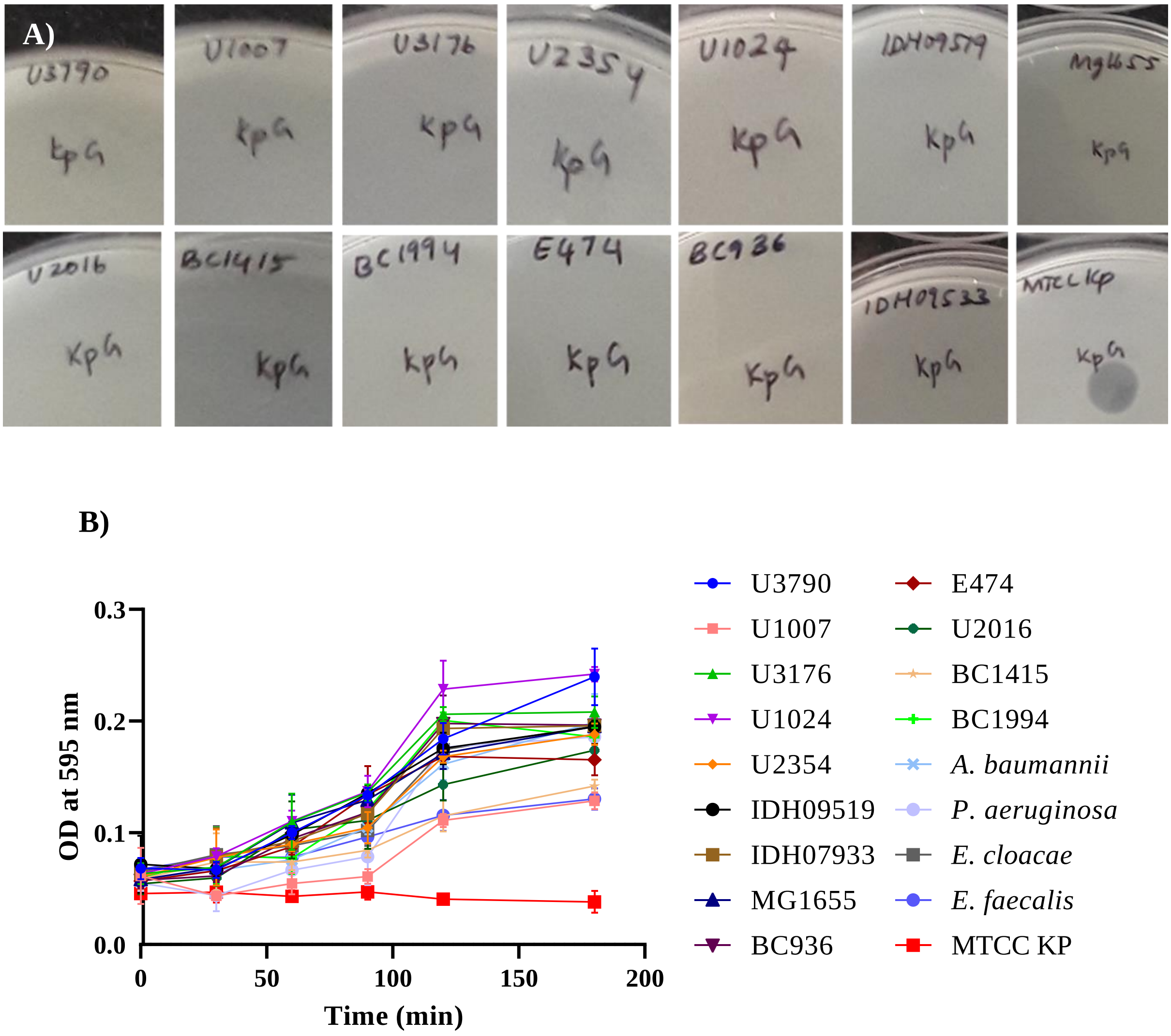

Fig. S4. One-step growth curve of KpG phage. KpG has a latent period of 25 min and a larger burst size of 224 PFU/ml.

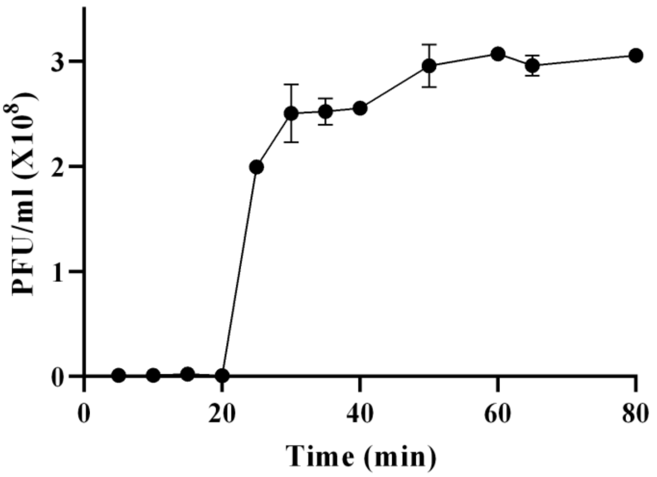

Fig. S5. PCR amplicons specific for *Podoviridae* from genomic DNA of KpG phage L 1 – 100bp ladder; L2 – PCR product of CL1; L3 – PCR product of CL2; L4-L6 – empty lanes.

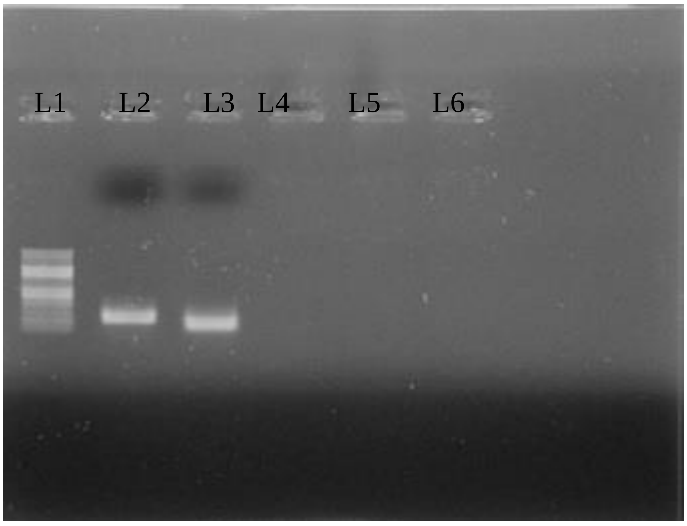

Fig. S6: Phage resistant strain of *K. pneumoniae* arises after 5 h post treatment with KpG. Phages at different dilutions were spotted on nutrient agar plate overlaid with (A) KpG unexposed and (B) exposed bacterial culture after 5 h. After overnight incubation at 37°C, the KpG exposed bacteria did not get lysed by KpG, whereas the unexposed group in similar condition got lysed as evident by the plaque formation.

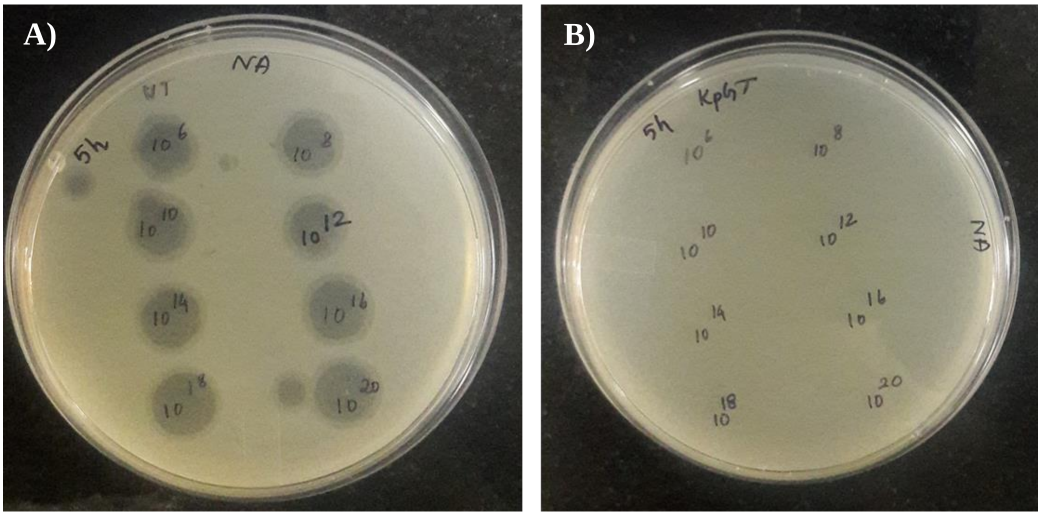

Fig. S7. **Live dead staining of biofilms treated with KpG phages.** Biofilms were formed on glass slides and treated with KpG. 24 h post treatment the slides were washed and stained with AO/PI and observed under Nikon fluorescent microscope.

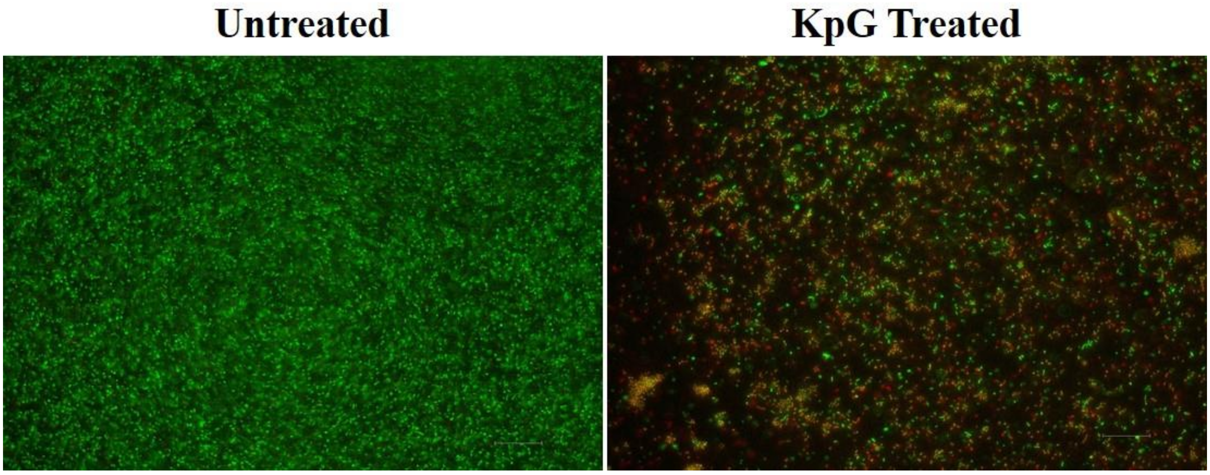

Fig. S8. Administration of KpG phages do not pose toxicity in zebrafish. KpG was injected intramuscularly in zebra fish. 48 h post injection, the fish were sacrificed and evaluated for their brain and liver enzyme profiles. Error bar represents the standard error of the mean. The difference in brain enzyme profile is not significant (P=0.0529).

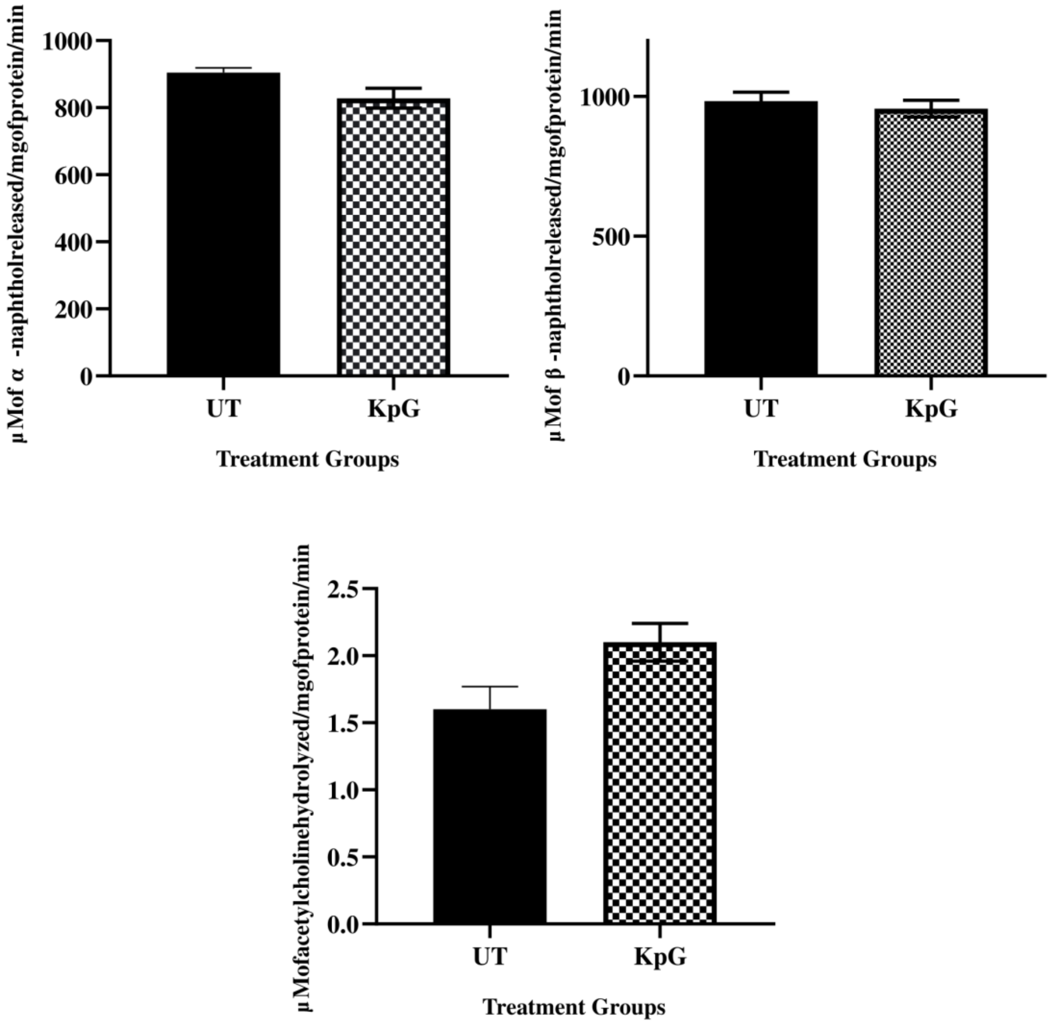

Fig. S9. Histopathological analysis of zebrafish injected intramuscularly with KpG phages. KpG was injected intramuscularly in zebrafish. 48 h post injection, the fish were sacrificed, preserved in formalin and embedded in paraffin wax. Tissue sections were made and stained with hematoxylin and eosin. Analysis of muscle and liver tissues of KpG injected fish did show significant immune response relative to untreated, depicting KpG's non-toxic nature in zebra fish.

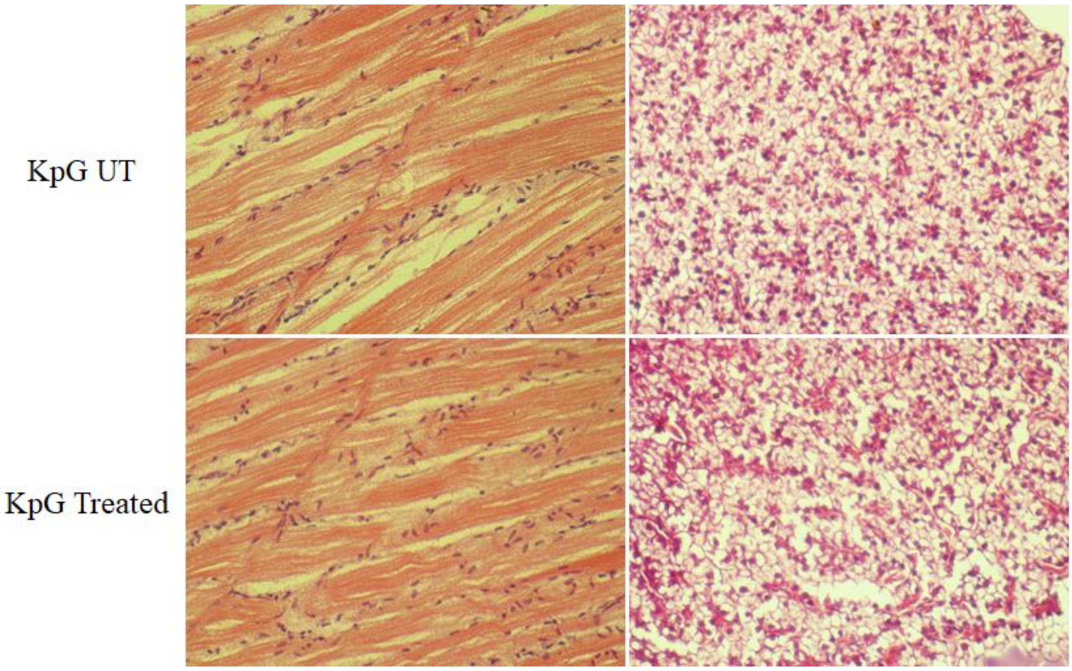

Supplement: Supplementary file 1 — Additional file 1. Electronic supplementary information. [file 13568_2021_1181_MOESM1_ESM.pdf]
